# Temporal Changes in Dispensing of Methylphenidate During Attention Deficit Hyperactivity Disorder Shortages in Australia: An Interrupted Time Series and Descriptive Analysis of National Dispensing Patterns

**DOI:** 10.1002/pds.70475

**Published:** 2026-09-08

**Authors:** George Gadalla, Wern Chai, Lisa Kalisch Ellett, Jack Janetzki

**Affiliations:** ^1^ School of Pharmacy and Biomedical Sciences, College of Health Adelaide University Adelaide South Australia Australia; ^2^ Health and Biomedical Innovation, College of Health Adelaide University Adelaide South Australia Australia; ^3^ Quality Use of Medicines and Pharmacy Research Centre, College of Health Adelaide University Adelaide South Australia Australia

**Keywords:** ADHD, medicine shortages, methylphenidate, pharmacoepidemiology, section 19A

## Abstract

**Purpose:**

To describe nationwide dispensing patterns of methylphenidate in Australia before and during recent prolonged supply shortages, and to assess the extent to which Section 19A (S19A) overseas substitutes contributed to population‐level utilisation during the shortage period. Secondary aims were to compare methylphenidate dispensing with other ADHD medicines, examine changes in dispensing across individual strengths and formulations and quantify delays between S19A approval and Pharmaceutical Benefits Scheme (PBS) listing.

**Methods:**

An interrupted time series (ITS) and descriptive analysis of population‐level dispensing patterns was conducted using publicly available monthly dispensing data from the Pharmaceutical Benefits Scheme (PBS) and Repatriation PBS (January 2020–March 2026). Data were standardised to dispensings and defined daily doses (DDD) per 100 000 population. Methylphenidate shortages were identified using the Therapeutic Goods Administration Medicines Shortages database and shortage months were designated where there was at least 1 day of shortage in a month. S19A approvals, PBS listing and lapse dates were obtained from the S19A approvals database to determine delays to subsidised access.

**Results:**

Dispensing of ADHD medicines increased substantially from 2020 to 2025. Methylphenidate dispensing increased overall but plateaued from late 2024 onward, coinciding with widespread supply disruptions affecting multiple strengths and formulations. DDD ITS analysis demonstrated abrupt reductions in methylphenidate strengths (particularly 36 and 54 mg) during shortages, with partial compensatory increases for some other strengths. Fifteen overseas‐registered methylphenidate products received S19A approval; however, of the 14 listed on the PBS, time to PBS listing ranged from 83 to 166 days (median 162 days). After PBS listing, S19A products contributed to 1.3% of all methylphenidate dispensings between July 2025 and March 2026.

**Conclusion:**

Methylphenidate dispensing in Australia became unstable during extensive supply shortages from late 2024. Although numerous S19A products were approved, there was minimal uptake of S19A PBS‐listed products at the population level. As ADHD medicine utilisation continues to rise, strengthened national strategies to improve supply chain resilience, transparency and responsiveness are required.

## Key Points

- Dispensing of ADHD medicines in Australia has increased substantially since 2020, reflecting rising demand and diagnosis.
- Methylphenidate supply shortages from late 2024 were associated with instability in dispensing, particularly for modified‐release formulations.
- Patterns in aggregate dispensing data were consistent with incomplete substitution across strengths and formulations.
- Although multiple overseas products were approved under Section 19A, delays to PBS listing occurred.
- Uptake of Section 19A products remained minimal, highlighting gaps in the responsiveness of current regulatory and subsidy pathways during shortages.

## Plain Language Summary

Use of medicines to treat attention deficit hyperactivity disorder (ADHD) has increased rapidly in Australia in recent years. This study examined how national dispensing patterns of methylphenidate changed before and during recent supply shortages. We found that while overall use continued to rise, dispensing became unstable during shortages, particularly for commonly used modified‐release forms which may have affected continuity of treatment. Although overseas versions of methylphenidate were approved for use in Australia to help address shortages, there were delays before these medicines were subsidised and very few were ultimately used. These findings suggest that current systems for managing medicine shortages may not respond quickly enough to support patients. As demand for ADHD medicines continues to grow, improved strategies are needed to ensure reliable access to treatment.

## 1 Purpose

Attention Deficit Hyperactivity Disorder (ADHD) is a chronic neurodevelopmental condition affecting attention, impulse control and activity regulation [1]. ADHD affects 6%–10% of Australian children and 3%–5% of adults [2]. Psychostimulants including methylphenidate, dexamfetamine and lisdexamfetamine are first‐line pharmacotherapy according to Australian Therapeutic Guidelines [1]. Other medicines used for ADHD in Australia are atomoxetine and guanfacine; however, these are not regarded as stimulants. Each of these medicines is listed specifically on Australia's national medicines formulary, the Pharmaceutical Benefits Scheme (PBS), for the management of ADHD. Clonidine, a non‐stimulant, which is also used for treatment of ADHD, is not specifically listed on the PBS for this indication. Whether a person can access subsidised medicines via the PBS is dependent on whether they were diagnosed with ADHD as a child or an adult [2]. For example, any patient with ADHD can receive methylphenidate, conventional release 10 mg tablets subsidised via the PBS, whereas to be eligible for PBS subsidy of Concerta modified release tablets, the patient needs to have been diagnosed with ADHD between the ages of 6 and 18 years. For other modified release methylphenidate brands, a patient is eligible if their ADHD was diagnosed between ages 6 and 17 years inclusive, or if they were diagnosed before turning 18 years and are continuing treatment after 18 years of age. Patients diagnosed retrospectively (confirmed to have had ADHD in childhood) are also eligible, whether they are starting or continuing PBS‐subsidised treatment after turning 18 years of age.

In October 2024, the sponsor of Concerta notified the Therapeutic Goods Administration (TGA) (Australia's national medicines regulator) of shortages affecting multiple modified release strengths due to manufacturing issues [3]. Shortages also affected Ritalin LA, while increased demand contributed to shortages of alternative brands such as Rubifen LA [3].

In Australia, medicines that can be legally supplied can be found in the Australian Register of Therapeutic Goods (ARTG). During periods of shortages, Section 19A (S19A), a provision of Australia's Therapeutic Goods Act, allows the temporary import and supply of unapproved products in order to mitigate impacts of shortages of approved products that appear on the ARTG [4, 5, 6]. PBS listing of S19A products is not automatic and the medicine sponsor must apply for PBS approval. There is usually a lag period between approval and listing of a product on the PBS.

Given prolonged methylphenidate shortages in 2024 and 2025, this study aimed to describe national dispensing patterns before and during these disruptions.

## 2 Methods

This population‐based interrupted time series and descriptive analysis of national dispensing patterns examined dispensing patterns of methylphenidate in Australia before and during shortages. Secondary objectives were to (1) compare methylphenidate dispensing with other ADHD medicines, (2) assess changes by strength or formulation and (3) quantify the proportion of methylphenidate dispensed as overseas imports (S19A) during shortages. The TGA Medicine Shortage Reports database was used to identify active and resolved methylphenidate shortages throughout the study period [3]. A calendar month was designated as a shortage month if there was a shortage notification active for at least 1 day during the month.

Dispensing data were obtained from the PBS and RPBS (Repatriation Pharmaceutical Benefits Scheme) Section 85 and 100 Date of Supply reports, which provide aggregated monthly dispensings by PBS item code [7]. Medicines included methylphenidate, dexamfetamine, lisdexamfetamine, atomoxetine, and guanfacine. Data were mapped to the PBS drug map (June 2026 edition) for descriptive information on name, strength, formulation and pack size, covering January 2020–March 2026.

Population‐standardised dispensings per 100 000 each month were calculated for each medicine using Australian Bureau of Statistics Estimated Residential Population data [8]. For methylphenidate, calculations were performed by strength and formulation (tablet, capsule, conventional, modified or extended release) and standardised to defined daily dose (DDD) per World Health Organisation methodology [9], combining total prescription counts for both ARTG and S19A PBS‐listed products:

$$\begin{array}{c} \mathrm{DDD}\;\mathrm{per}\;100,000\;\text{population}\;\mathrm{per}\;\mathrm{day}=\frac{\text{Strength of formulation}\;\left(\mathrm{mg}\right)\times \text{pack size}\times \text{prescriptions dispensed}\;\mathrm{per}\;\text{month}\times 100,000}{\mathrm{DDD}\;\left(\text{from}\;\mathrm{WHO}\;\text{database}\right)\times \text{population}\times \text{days in month}} \end{array}$$

DDDs were expressed per 100 000 population per day for each month of the study period because the DDD is the assumed average maintenance dose per day of a medicine when used for its main indication in adults. Expressing the DDD in this way allows for monitoring population‐level medicine use during periods of medicine shortages. A reduction in DDD volume may be consistent with treatment interruption for some patients, although alternative explanations cannot be excluded. For the pack size in the DDD calculation above, all brands of ARTG‐registered and S19A listed modified‐release methylphenidate products in this study were available in packs containing 30 units (tablets or capsules) [10, 11]. For this study, for simplicity, extended‐release formulations of methylphenidate are referred to as modified‐release. It is also important to note that the Ritalin brand and its generic equivalents are not considered interchangeable on the PBS with Concerta and its generic equivalents. The conventional‐release methylphenidate 10 mg tablets were only available in a pack size of 100 tablets regardless of brand at the time of the study [10]. We assumed that the quantity supplied with each dispensing was one full pack size.

To assess the proportion of monthly dispensings attributable to S19A overseas imports of methylphenidate, the number of dispensings was classified as Australian Register of Therapeutic Goods (ARTG)‐listed products or PBS‐listed S19A products, based on PBS item codes for these products.

The S19A Approvals database was also searched to provide information on specific products that received approval, the date of the approval, whether it was PBS listed and when S19A approval lapsed [4, 11]. This data was then used to calculate the number of days between S19A approval and PBS listing. To determine the percentage of methylphenidate dispensings attributable to S19A products from first listing in July to March 2026, we divided the total number of S19A dispensings by ARTG‐listed dispensings across this period and multiplied this by 100.

An interrupted time series (ITS) was conducted using the DDD/100000 population per day at the individual product strength level to evaluate the impact of the shortage on utilisation of methylphenidate before and after the first month of sustained shortages which was September 2024. Linear regression models were used to estimate the change in dispensing rates per 100 000 population in the 56 months before and 18 months after September 2024 to March 2025, adjusting for seasonality and autocorrelation [12]. September 2024, as the intervention month, was treated as a transition month, and given that the shortages began partway through the month, this month was not included in the models [12]. SAS on Demand for Academics was used for the ITS analysis.

This study used publicly and freely available aggregate dispensing data; therefore, ethics approval was not required. This study was conducted and reported in accordance with the RECORD‐PE guidelines.

## 3 Results

Multiple methylphenidate supply interruptions were reported between 2020 and 2026, affecting a range of methylphenidate brands, strengths and formulations, including modified‐release capsules and tablets (Table 1). Reasons included manufacturing issues, transport/logistics/storage capacity constraints and unexpected increases in demand. Several modified release Concerta strengths were subsequently listed as in shortage from late 2024, with prolonged manufacturing‐related supply disruptions extending into 2026 (Table 1).

**TABLE 1:** Methylphenidate shortages from January 2020 onwards from the Therapeutic Goods Administration Medicines Shortages database.

ARTG name	Sponsor	Start date	End date	Reason
RITALIN LA methylphenidate hydrochloride 10 mg modified‐release capsule bottle	Novartis Pharmaceuticals Australia Pty Ltd	4/02/2020	16/03/2020	Other
RITALIN 10 methylphenidate hydrochloride 10 mg tablet blister pack	Novartis Pharmaceuticals Australia Pty Ltd	5/02/2020	16/03/2020	Other
ARTIGE methylphenidate hydrochloride 10 mg tablet blister pack	Novartis Pharmaceuticals Australia Pty Ltd	7/02/2020	16/03/2020	Other
RITALIN LA methylphenidate hydrochloride 30 mg modified release capsule bottle (NF)	Novartis Pharmaceuticals Australia Pty Ltd	8/02/2020	16/03/2020	Other
RITALIN LA methylphenidate hydrochloride 20 mg modified release capsule bottle (NF)	Novartis Pharmaceuticals Australia Pty Ltd	13/02/2020	16/03/2020	Other
RITALIN LA methylphenidate hydrochloride 40 mg modified release capsule bottle (NF)	Novartis Pharmaceuticals Australia Pty Ltd	15/02/2020	16/03/2020	Other
RITALIN 10 methylphenidate hydrochloride 10 mg tablet blister pack	Novartis Pharmaceuticals Australia Pty Ltd	16/02/2021	1/03/2021	Other
ARTIGE methylphenidate hydrochloride 10 mg tablet blister pack	Novartis Pharmaceuticals Australia Pty Ltd	16/02/2021	1/03/2021	Other
ARTIGE methylphenidate hydrochloride 10 mg tablet blister pack	Novartis Pharmaceuticals Australia Pty Ltd	8/09/2021	17/09/2021	Manufacturing
RITALIN LA methylphenidate hydrochloride 60 mg modified release capsule bottle	Novartis Pharmaceuticals Australia Pty Ltd	26/04/2022	28/04/2022	Transport/Logistic issues/Storage capacity issues
RITALIN LA methylphenidate hydrochloride 10 mg modified‐release capsule bottle	Novartis Pharmaceuticals Australia Pty Ltd	26/05/2022	4/06/2022	Manufacturing
RITALIN LA methylphenidate hydrochloride 60 mg modified release capsule bottle	Novartis Pharmaceuticals Australia Pty Ltd	15/07/2022	28/07/2022	Manufacturing
RITALIN LA methylphenidate hydrochloride 20 mg modified release capsule bottle (NF)	Novartis Pharmaceuticals Australia Pty Ltd	15/07/2022	16/08/2022	Manufacturing
RITALIN LA methylphenidate hydrochloride 30 mg modified release capsule bottle (NF)	Novartis Pharmaceuticals Australia Pty Ltd	15/07/2022	16/08/2022	Manufacturing
RITALIN LA methylphenidate hydrochloride 40 mg modified release capsule bottle (NF)	Novartis Pharmaceuticals Australia Pty Ltd	15/07/2022	8/08/2022	Manufacturing
RITALIN LA methylphenidate hydrochloride 10 mg modified‐release capsule bottle	Novartis Pharmaceuticals Australia Pty Ltd	18/07/2022	16/08/2022	Manufacturing
ARTIGE methylphenidate hydrochloride 10 mg tablet blister pack	Novartis Pharmaceuticals Australia Pty Ltd	30/11/2022	9/12/2022	Transport/Logistic issues/Storage capacity issues
METHYLPHENIDATE XR ARX methylphenidate hydrochloride 36 mg modified release tablet bottle	Teva Pharma Australia Pty Ltd	12/07/2023	1/09/2023	Unexpected increase in consumer demand
METHYLPHENIDATE XR ARX methylphenidate hydrochloride 27 mg modified release tablet bottle	Teva Pharma Australia Pty Ltd	20/02/2024	22/05/2024	Transport/Logistic issues/Storage capacity issues
RITALIN LA methylphenidate hydrochloride 60 mg modified release capsule bottle	Novartis Pharmaceuticals Australia Pty Ltd	30/04/2024	8/05/2024	Manufacturing
CONCERTA methylphenidate hydrochloride 18 mg extended release tablet bottle	Janssen‐Cilag Pty Ltd	21/06/2024	2/07/2024	Manufacturing
CONCERTA methylphenidate hydrochloride 54 mg extended release tablet bottle	Janssen‐Cilag Pty Ltd	9/07/2024	16/07/2024	Manufacturing
CONCERTA methylphenidate hydrochloride 36 mg extended release tablet bottle	Janssen‐Cilag Pty Ltd	14/08/2024	20/08/2024	Manufacturing
CONCERTA methylphenidate hydrochloride 18 mg extended release tablet bottle	Janssen‐Cilag Pty Ltd	28/08/2024	25/09/2024	Manufacturing
METHYLPHENIDATE‐TEVA XR methylphenidate hydrochloride 18 mg modified release tablet bottle	Teva Pharma Australia Pty Ltd	4/09/2024	15/09/2025	Unexpected increase in demand due to other sponsors unable to supply
METHYLPHENIDATE‐TEVA XR methylphenidate hydrochloride 54 mg modified release tablet bottle	Teva Pharma Australia Pty Ltd	30/09/2024	14/10/2025	Unexpected increase in demand due to other sponsors unable to supply
METHYLPHENIDATE‐TEVA XR methylphenidate hydrochloride 27 mg modified release tablet bottle	Teva Pharma Australia Pty Ltd	11/10/2024	11/10/2024	Manufacturing
CONCERTA methylphenidate hydrochloride 54 mg modified release tablet bottle	Janssen‐Cilag Pty Ltd	16/10/2024	14/04/2026	Manufacturing
CONCERTA methylphenidate hydrochloride 18 mg modified release tablet bottle	Janssen‐Cilag Pty Ltd	29/10/2024	14/04/2026	Manufacturing
RITALIN LA methylphenidate hydrochloride 40 mg modified release capsule bottle (NF)	Novartis Pharmaceuticals Australia Pty Ltd	15/11/2024	17/12/2024	Transport/Logistic issues/Storage capacity issues
RITALIN LA methylphenidate hydrochloride 60 mg modified release capsule bottle	Novartis Pharmaceuticals Australia Pty Ltd	25/11/2024	28/11/2024	Transport/Logistic issues/Storage capacity issues
CONCERTA methylphenidate hydrochloride 36 mg modified release tablet bottle	Janssen‐Cilag Pty Ltd	16/12/2024	14/04/2026	Manufacturing
RITALIN 10 methylphenidate hydrochloride 10 mg tablet blister pack	Novartis Pharmaceuticals Australia Pty Ltd	18/12/2024	19/12/2024	Manufacturing
ARTIGE methylphenidate hydrochloride 10 mg tablet blister pack	Novartis Pharmaceuticals Australia Pty Ltd	18/12/2024	19/12/2024	Manufacturing
CONCERTA methylphenidate hydrochloride 27 mg modified release tablet bottle	Janssen‐Cilag Pty Ltd	19/12/2024	14/04/2026	Manufacturing
RITALIN LA methylphenidate hydrochloride 30 mg modified release capsule bottle (NF)	Novartis Pharmaceuticals Australia Pty Ltd	23/12/2024	6/01/2025	Manufacturing
METHYLPHENIDATE‐TEVA XR methylphenidate hydrochloride 36 mg modified release tablet bottle	Teva Pharma Australia Pty Ltd	14/01/2025	18/07/2025	Unexpected increase in demand due to other sponsors unable to supply
METHYLPHENIDATE‐TEVA XR methylphenidate hydrochloride 27 mg modified release tablet bottle	Teva Pharma Australia Pty Ltd	14/01/2025	14/10/2025	Unexpected increase in demand due to other sponsors unable to supply
RITALIN LA methylphenidate hydrochloride 40 mg modified release capsule bottle (NF)	Novartis Pharmaceuticals Australia Pty Ltd	14/01/2025	24/01/2025	Unexpected increase in consumer demand
RITALIN LA methylphenidate hydrochloride 40 mg modified release capsule bottle (NF)	Novartis Pharmaceuticals Australia Pty Ltd	26/03/2025	26/03/2025	Manufacturing
RITALIN 10 methylphenidate hydrochloride 10 mg tablet blister pack	Novartis Pharmaceuticals Australia Pty Ltd	7/04/2025	22/04/2025	Transport/Logistic issues/Storage capacity issues
ARTIGE methylphenidate hydrochloride 10 mg tablet blister pack	Novartis Pharmaceuticals Australia Pty Ltd	7/04/2025	15/04/2025	Transport/Logistic issues/Storage capacity issues
RUBIFEN LA methylphenidate hydrochloride 60 mg modified release capsules blister pack	AFT Pharmaceuticals Pty Ltd	18/04/2025	1/09/2026	Unexpected increase in demand due to other sponsors unable to supply
RITALIN LA methylphenidate hydrochloride 40 mg modified release capsule bottle	Novartis Pharmaceuticals Australia Pty Ltd	29/04/2025	27/03/2026	Manufacturing
RUBIFEN LA methylphenidate hydrochloride 40 mg modified release capsules blister pack	AFT Pharmaceuticals Pty Ltd	13/05/2025	1/09/2026	Unexpected increase in demand due to other sponsors unable to supply
RUBIFEN LA methylphenidate hydrochloride 10 mg modified release capsules blister pack	AFT Pharmaceuticals Pty Ltd	3/06/2025	1/09/2026	Unexpected increase in demand due to other sponsors unable to supply
RUBIFEN LA methylphenidate hydrochloride 30 mg modified release capsules blister pack	AFT Pharmaceuticals Pty Ltd	10/06/2025	1/09/2026	Unexpected increase in demand due to other sponsors unable to supply
RUBIFEN LA methylphenidate hydrochloride 20 mg modified release capsules blister pack	AFT Pharmaceuticals Pty Ltd	28/07/2025	1/09/2026	Unexpected increase in demand due to other sponsors unable to supply
RITALIN 10 methylphenidate hydrochloride 10 mg tablet blister pack	Novartis Pharmaceuticals Australia Pty Ltd	28/08/2025	28/08/2025	Manufacturing

Fifteen overseas registered methylphenidate products received S19A approval during the study period. Fourteen of these were subsequently listed on the PBS. Overall, for the 14 S19A products listed on the PBS, time from S19A approval to PBS listing ranged from 83 to 166 days, with a median time of 162 days (IQR 104–166) indicating that even at the 25th percentile, delays exceeded 3 months (Table 2).

**TABLE 2:** Section 19A approved medicines by product, S19A approval date, PBS listing date, lapse date (where known) and the number of days between S19A approval and PBS listing.

Section 19A medicine	S19A approval date	Date PBS listed	S19A approval lapse date	Days to PBS listing
Concerta (Switzerland) Tablet containing methylphenidate hydrochloride 18 mg (extended release) Concerta (Switzerland) (s19A)	16/01/2025	1/07/2025	31/01/2026	166
Concerta (Switzerland) Tablet containing methylphenidate hydrochloride 27 mg (extended release), (s19A)	16/01/2025	1/07/2025	31/01/2026	166
Concerta (Switzerland) Tablet containing methylphenidate hydrochloride 36 mg (extended release) Concerta (Switzerland) (s19A)	16/01/2025	1/07/2025	31/01/2026	166
Concerta (Switzerland) Tablet containing methylphenidate hydrochloride 54 mg (extended release) Concerta (Switzerland) (s19A)	16/01/2025	1/07/2025	31/01/2026	166
Methylphenidate Orifarm 10 mg (Sweden) Capsule containing methylphenidate hydrochloride 10 mg (modified release), (s19A)	20/05/2025	1/09/2025	31/01/2026	104
Methylphenidate Orifarm 20 mg (Sweden) Capsule containing methylphenidate hydrochloride 20 mg (modified release), (s19A)	20/05/2025	1/09/2025	31/01/2026	104
Methylphenidate Orifarm 30 mg (Sweden) Capsule containing methylphenidate hydrochloride 30 mg (modified release), (s19A)	20/05/2025	1/09/2025	31/01/2026	104
Methylphenidate Orifarm 60 mg (Denmark) Capsule containing methylphenidate hydrochloride 60 mg (modified release), (s19A)	20/05/2025	1/09/2025	31/01/2026	104
Ritalin LA 10 mg (Switzerland) Capsule containing methylphenidate hydrochloride 10 mg (modified release) (Switzerland) (S19A)	23/05/2025	1/11/2025	30/04/2026	162
Ritalin LA 20 mg (Switzerland) Capsule containing methylphenidate hydrochloride 20 mg (modified release) (Switzerland) (s19A)	23/05/2025	1/11/2025	30/04/2026	162
Ritalin LA 30 mg (Switzerland) Capsule containing methylphenidate hydrochloride 30 mg (modified release) (Switzerland) (s19A)	23/05/2025	1/11/2025	30/04/2026	162
Ritalin LA 40 mg (Switzerland) Capsule containing methylphenidate hydrochloride 40 mg (modified release) (Switzerland) (s19A)	23/05/2025	1/11/2025	30/04/2026	162
Methylphenidate Hydrochloride Extended‐Release Capsules (LA) 60 mg (Granules Pharmaceuticals Inc., USA)	19/06/2025	Not PBS listed and not observable in dispensing data in this study	31/01/2026	Not PBS listed and not observable in dispensing data in this study
Atenza (Spain) Tablet containing methylphenidate hydrochloride 27 mg (modified release) (Spain) (S19A)	10/10/2025	1/01/2026	31/01/2026	83
Atenza (Spain) Tablet containing methylphenidate hydrochloride 54 mg (modified release) (Spain) (S19A)	10/10/2025	1/01/2026	31/01/2026	83

Despite PBS listing, S19A products contributed minimally (< 1.3%) to total methylphenidate dispensing between July 2025 and March 2026 (July 2025: 0.12%; greatest amount in November 2025: 3.26%; March 2026: 1.03%).

### 3.1 Overall

From January 2020 to March 2026, the population‐adjusted dispensing rate of ADHD medicines in Australia increased for most medicines included in the analysis. Lisdexamfetamine demonstrated the largest growth, rising from approximately 100 dispensings/100000 population/month in January 2020 to over 1000 dispensings/100000 population/month by March 2026. Methylphenidate dispensing also increased overall, from approximately 270 per 100 000 population/month in early 2020 to approximately 750 per 100 000 population/month by March 2026.

Dexamfetamine dispensing increased across the study period from approximately 115 to 450/100000 population/month, as did guanfacine from approximately 50 to over 250/100000 population/day. Atomoxetine was dispensed at low volume and demonstrated modest growth throughout the study period.

**FIGURE 1:**
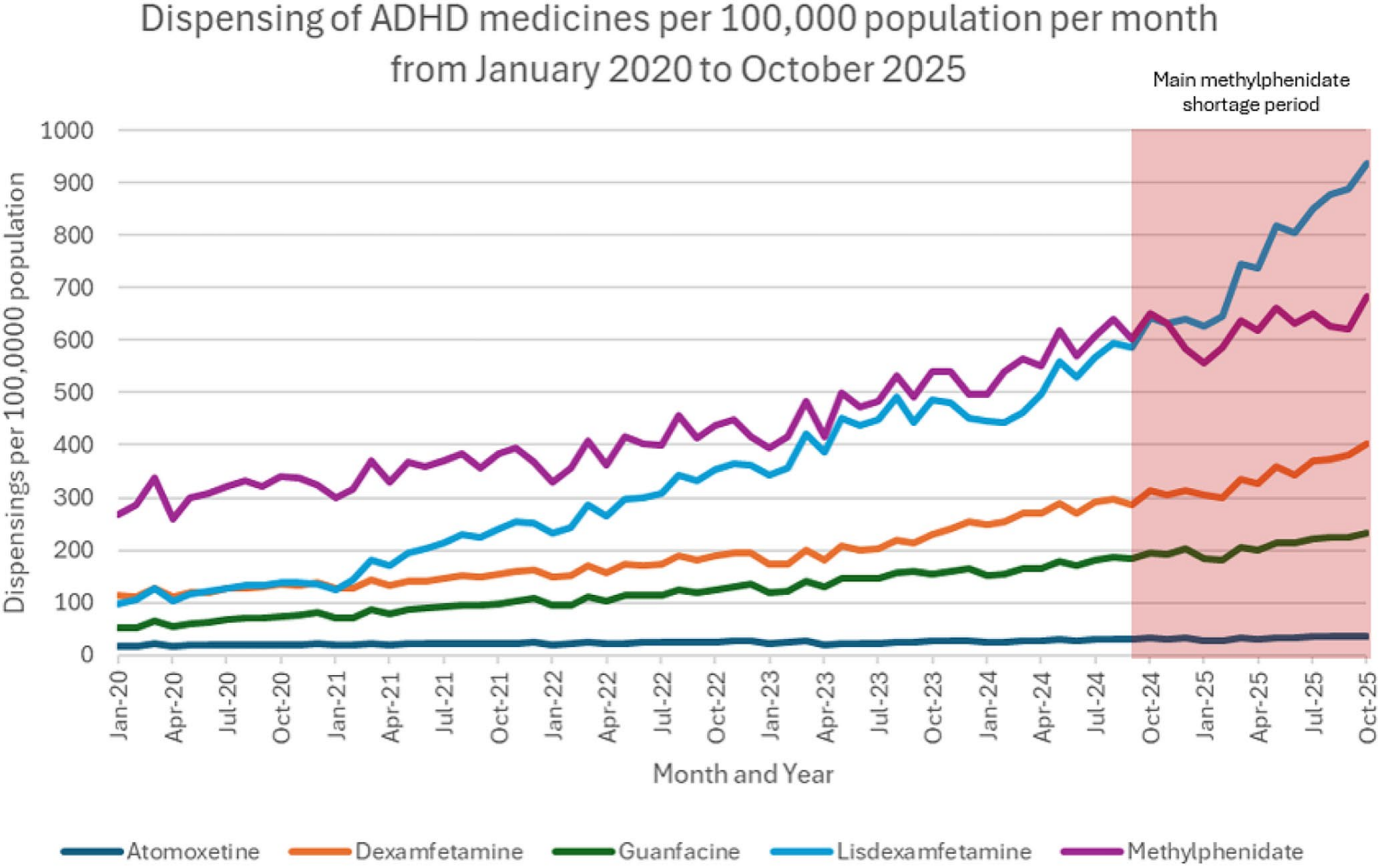
Dispensings of ADHD medicines in Australia per 100 000 population per month from January 2020 to March 2026.

From late 2024 to early 2026, methylphenidate dispensing plateaued, while lisdexamfetamine continued to rise, resulting in lisdexamfetamine becoming the most frequently dispensed ADHD medicine from November 2024 onwards (Figure 1).

**FIGURE 2:**
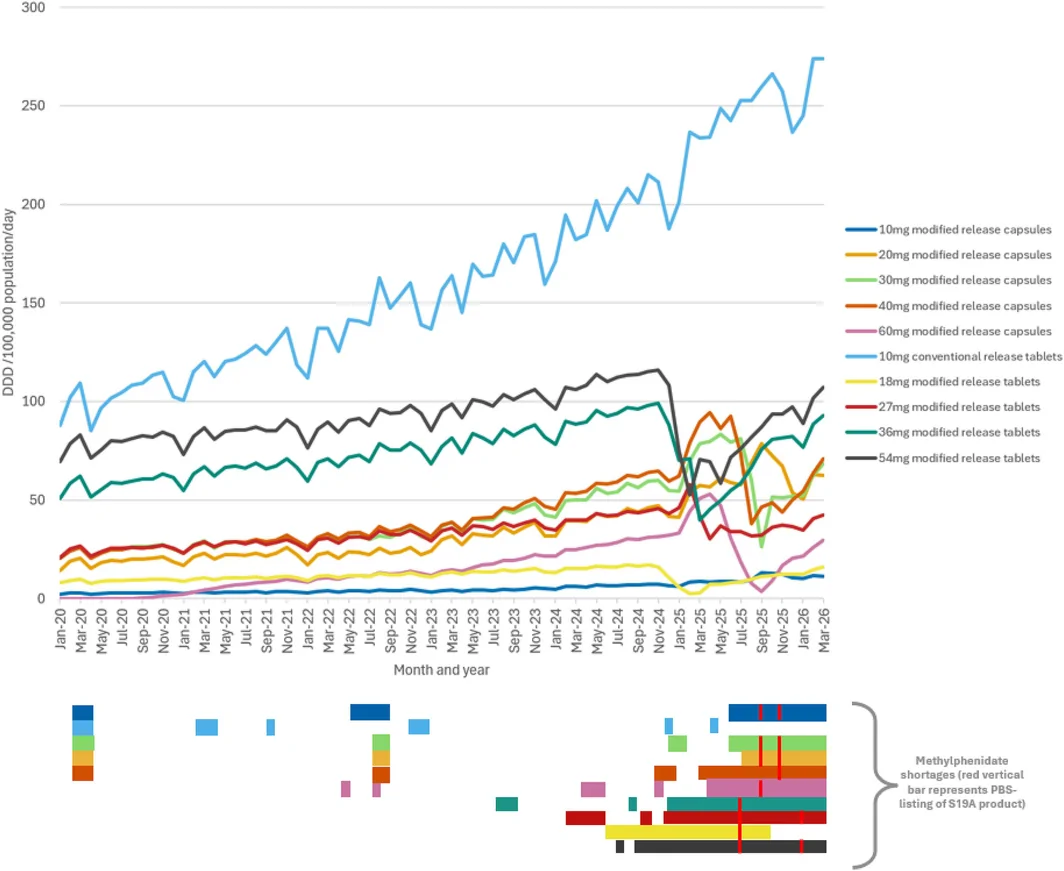
Defined Daily Dose per 100 000 population per day of methylphenidate by strength and formulation from January 2020 to March 2026. Shortage periods are highlighted in coloured bars in the lower panel.

DDD analysis of methylphenidate dispensings/100000 population/day (Figure 2) demonstrated sustained growth in the volume of use of both conventional and modified release methylphenidate products from 2020 to 2024.

In early 2025, there were abrupt declines in DDD/100000 population/day for several methylphenidate products corresponding with reported supply disruptions. Higher‐strength modified‐release tablets (36 and 54 mg) showed marked reductions in DDD/100000 population/day during 2025, while some lower strengths of modified‐release capsules such as the 20, 40 and 30 mg capsules demonstrated compensatory increases (Figure 2).

Conventional 10 mg tablets increased gradually across the study period but did not fully offset reductions observed in modified‐release products during 2025.

### 3.2 Methylphenidate Capsules Versus Tablets

Between 2020 and 2024, dispensing rates of both capsule and tablet formulations of methylphenidate increased. Tablets consistently accounted for higher dispensing rates per 100 000 population per month than capsules throughout the study period. Tablet dispensings rose from approximately 200 dispensings/100000 population/month in early 2020 to over 400 dispensings/100000 population/month by late 2024. Capsules increased from approximately 70 dispensings/100000 population/month to approximately 200 dispensings/100000 population/month over the same period.

**FIGURE 3:**
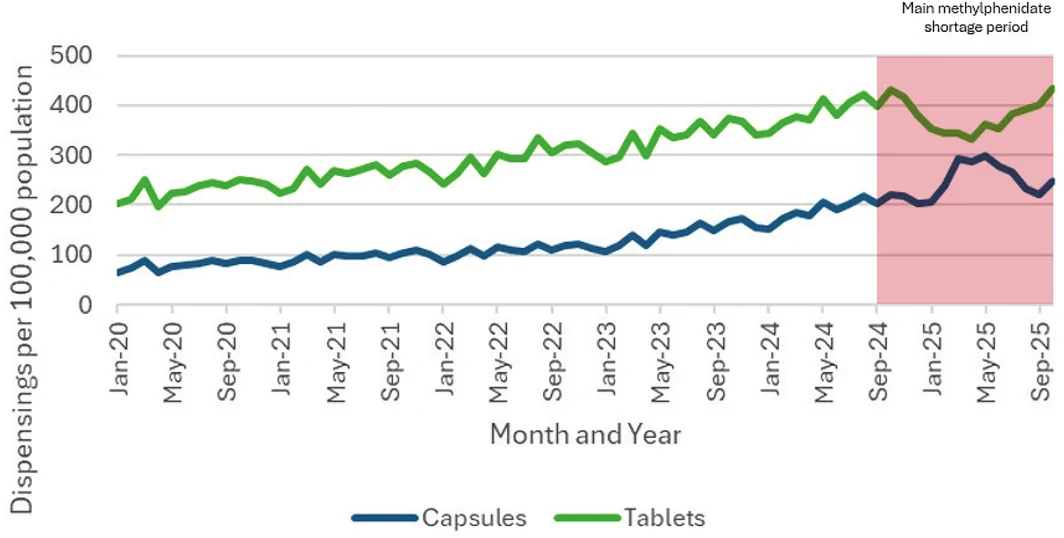
Dispensings of methylphenidate capsules or tablets from January 2020 to March 2026.

In early months of 2025, methylphenidate capsule dispensings demonstrated a marked increase, followed by volatility and a subsequent decline mid‐year before partial recovery by October 2025. Methylphenidate tablet dispensings showed a noticeable decline in early 2025, with gradual recovery towards the end of the observation period (Figure 3).

### 3.3 Interrupted Time Series Analysis

Table 3 describes the interrupted time series statistics for each methylphenidate product strength and formulation. The single conventional release 10 mg product, and each strength of modified release tablet or capsule are presented in the table.

**TABLE 3:** Interrupted time series statistics for each methylphenidate product strength and formulation.

Conventional 10 mg tablet				
Term	Total *R* ^2^	DDD/100000/day	SE	*p*
DDD/100000/day in Jan 2020	0.9829	86.6444	3.3875	< 0.0001
Monthly change pre‐Sep 2024		1.8627	0.0791	< 0.0001
Change in Sep 2024		8.9979	4.4847	0.0497
Monthly change post‐Sep 2024		1.8880	0.3640	< 0.0001

10 mg modified release				
Term	Total *R* ^2^	DDD/100000/day	SE	*p*
DDD/100000/day in Jan 2020	0.9674	2.1028	0.3144	< 0.0001
Monthly change pre‐Sep 2024		0.0645	0.008599	< 0.0001
Change in Sep 2024		0.1247	0.5055	0.8061
Monthly change post‐Sep 2024		0.2746	0.0452	< 0.0001

20 mg modified release				
Term	Total *R* ^2^	DDD/100000/day	SE	*p*
DDD/100000/day in Jan 2020	0.9661	13.1642	2.2402	< 0.0001
Monthly change pre‐Sep 2024		0.4540	0.0617	< 0.0001
Change in Sep 2024		4.5115	2.9371	0.1303
Monthly change post‐Sep 2024		0.8044	0.2774	0.0054

30 mg modified release				
Term	Total *R* ^2^	DDD/100000/day	SE	*p*
DDD/100000/day in Jan 2020	0.8974	19.6172	2.8238	< 0.0001
Monthly change pre‐Sep 2024		0.5461	0.0695	< 0.0001
Change in Sep 2024		21.8198	4.7998	< 0.0001
Monthly change post‐Sep 2024		−1.4179	0.3920	0.0006

40 mg modified release				
Term	Total *R* ^2^	DDD/100000/day	SE	*p*
DDD/100000/day in Jan 2020	0.9318	17.4715	2.9969	< 0.0001
Monthly change pre‐Sep 2024		0.6537	0.0751	< 0.0001
Change in Sep 2024		22.2504	4.6481	< 0.0001
Monthly change post‐Sep 2024		−1.6682	0.4032	0.0001

60 mg modified release				
Term	Total *R* ^2^	DDD/100000/day	SE	*p*
DDD/100000/day in Jan 2020	0.9618	−5.0698	1.3330	0.0004
Monthly change pre‐Sep 2024		0.5431	0.0317	< 0.0001
Change in Sep 2024		14.9134	2.3443	< 0.0001
Monthly change post‐Sep 2024		−1.7939	0.2029	< 0.0001

18 mg extended release				
Term	Total *R* ^2^	DDD/100000/day	SE	*p*
DDD/100000/day in Jan 2020	0.8587	7.9577	0.5223	< 0.0001
Monthly change pre‐Sep 2024		0.1276	0.0139	< 0.0001
Change in Sep 2024		−5.2083	1.2079	< 0.0001
Monthly change post‐Sep 2024		−0.0840	0.1038	0.4217

27 mg extended release				
Term	Total *R* ^2^	DDD/100000/day	SE	*p*
DDD/100000/day in Jan 2020	0.8800	22.4311	1.1059	< 0.0001
Monthly change pre‐Sep 2024		0.3474	0.0281	< 0.0001
Change in Sep 2024		4.0753	2.2244	0.0724
Monthly change post‐Sep 2024		−0.9405	0.1820	< 0.0001

36 mg extended release				
Term	Total *R* ^2^	DDD/100000/day	SE	*p*
DDD/100000/day in Jan 2020	0.8558	57.8838	2.5069	< 0.0001
Monthly change pre‐Sep 2024		0.6674	0.0640	< 0.0001
Change in Sep 2024		−16.3743	5.6034	0.0050
Monthly change post‐Sep 2024		−0.7791	0.4732	0.1054

54 mg extended release				
Term	Total *R* ^2^	DDD/100000/day	SE	*p*
DDD/100000/day in Jan 2020	0.8431	80.3710	2.6697	< 0.0001
Monthly change pre‐Sep 2024		0.5831	0.0703	< 0.0001
Change in Sep 2024		−14.3420	5.7399	0.0155
Monthly change post‐Sep 2024		−1.0848	0.4937	0.0322

For the 10 mg conventional release methylphenidate tablets, there was an immediate and statistically significant change in September 2024 of 8.9979 DDD/100000/day (*p* < 0.001); however, the post‐September 2024 rate was similar to the rate prior to September 2024.

For the modified‐release methylphenidate capsules, there was a statistically significant change in dispensing rates in September only for the 30 mg, 40 mg, and 60 mg strengths with a rate of change between 14.91 and 22.25 DDD/100000/day, respectively. The change in dispensing rate post‐September 2024 was negative for the 30 mg, 40 mg, and 60 mg strengths and statistically significant (*p* < 0.05), indicating that these were the strengths most affected by the shortages.

For the modified‐release methylphenidate tablets, the change in dispensing rate in September 2024 was negative and statistically significant for all strengths except the 27 mg strength, although the positive change in rate for this strength was not statistically significant (*p* = 0.0724). There was a negative rate change for all strengths post‐September 2024, but this was only statistically significant for the 27 mg and 54 mg strengths, indicating that they were the most affected by the shortages.

## 4 Discussion

Dispensing of ADHD medicines in Australia increased between 2020 and 2025, with marked growth in supply of lisdexamfetamine and methylphenidate. From late 2024 into 2025, methylphenidate dispensings fluctuated considerably, coinciding with widespread and prolonged supply disruptions affecting multiple strengths and formulations. Although 14 Section 19A (S19A) alternatives were listed on the PBS across the shortage periods, their contribution to overall methylphenidate dispensing remained minimal (1.3% of monthly dispensings from July 2025 to March 2026), suggesting limited population‐level mitigation of shortages through this mechanism.

### 4.1 Rising Utilisation

ADHD medicine use increased substantially across the observation period, consistent with international trends [13]. Increasing diagnosis and pharmacological treatment of ADHD have been reported across North America, Europe and parts of Asia over the past decade [14, 15, 16, 17]. In particular, stimulant prescribing has risen among adolescents and adults, reflecting expanded recognition of adult ADHD and evolving treatment guidelines [15, 18].

In Australia, previous analyses have demonstrated steady increases in stimulant prescribing over time [19]. Our findings extend this literature by demonstrating that growth accelerated further between 2020 and 2026, with lisdexamfetamine becoming the most dispensed ADHD medicine by November 2024 [19]. Similar shifts towards modified‐release formulations have been described internationally, driven by convenience, improved adherence, and reduced diversion risk relative to immediate‐release products [14, 15, 17, 20].

It is possible that the marked growth in lisdexamfetamine dispensing may also reflect substitution during methylphenidate shortages, a pattern consistent with evidence that medicine shortages frequently lead to (and also recommendation of) therapeutic switching within the same class [19, 21, 22]. In our previous national analysis of lisdexamfetamine shortages in Australia (2022–2024), we observed substantial within‐medicine substitution across strengths during periods of constrained supply, with increases in higher‐strength products when 30 and 50 mg strengths were unavailable [19]. DDD patterns suggested prescribers and patients adjusted strengths to maintain treatment continuity. Together, these findings indicate that stimulant shortages may drive both intra‐product strength substitution and inter‐product switching within the stimulant class, reflecting adaptive prescribing and dispensing behaviours in response to supply instability. It is important to note, however, that use of aggregate monthly dispensing data in this study precludes analysis of new initiators of lisdexamfetamine, people switching from methylphenidate and those who require dose adjustments or who may engage in stockpiling during shortages. Given that dispensing of other ADHD medicines such as dexamfetamine and guanfacine increased throughout the observation period, it is likely that a substantial part of the lisdexamfetamine growth reflects the underlying secular trend in ADHD pharmacotherapy rather than methylphenidate shortage‐driven substitution.

### 4.2 Temporal Changes in Methylphenidate Utilisation due to Supply Disruptions

From late 2024 onward, dispensing patterns for methylphenidate, particularly modified‐release capsule and formulations, became irregular. These fluctuations temporally corresponded with numerous reported manufacturing, logistics and demand‐related supply disruptions across multiple sponsors and strengths [3, 22]. Medicine shortages are a global issue driven by manufacturing, supply chain, and demand factors [23, 24, 25, 26, 27] Similar stimulant shortages internationally have been associated with treatment disruption and switching [28, 29, 30, 31]. Our findings are consistent with this literature. Several modified‐release methylphenidate strengths showed abrupt reductions in defined daily dose during 2025, while other strengths or alternative ADHD medicines increased, suggesting the possibility of partial substitution. It is possible that people were swapping from the 27 or 36 mg modified‐release to the 30 mg modified‐release due to shortages, as indicated by the changes in DDD/100000 population/day in Figure 2. Increases in dispensing of several strengths of methylphenidate did not fully offset reductions in disrupted products, indicating incomplete therapeutic substitution at the population level.

Such instability may have important clinical implications. Disruptions in ADHD pharmacotherapy are associated with symptom relapse, impaired functioning and reduced adherence [28]. Medicine shortages have also been linked to medication errors, increased healthcare utilisation and patient distress [21, 32, 33].

### 4.3 Limited Utilisation of S19A‐Listed Alternatives

A key finding of this study was the delay between S19A approval and PBS listing (~3 to 5.5 months). While S19A approvals enable legal importation and supply of substitute products during shortages, timely PBS listing is required to ensure subsidised access at scale. This lag indicates that regulatory approval does not necessarily translate into immediate affordability or widespread uptake, potentially limiting the short‐term utilisation of S19A measures during acute supply disruptions. Consistent with this, our analysis showed that even after PBS listing of 14 of the 15 S19A approved methylphenidate products, S19A alternatives accounted for less than 1.5% of total methylphenidate dispensings between July and March 2026, suggesting that importation played only a minor role in mitigating the shortage at a population level.

These findings contrast with our previous national evaluation of 15 PBS‐listed S19A medicines (2016–2024), in which S19A products accounted for more than half of total dispensing volume within 1 year of PBS listing for most medicines examined [5]. Together, the findings of our previous study and the current findings suggest that the effectiveness of the S19A pathway is context dependent. In high‐demand therapeutic areas such as ADHD, where shortages affect multiple strengths and sponsors simultaneously, S19A importation may be insufficient to stabilise utilisation at the population level.

Internationally, regulatory mechanisms to manage shortages (including emergency provision of medicines, importation pathways, expedited approvals, and therapeutic substitution policies) vary in effectiveness [5, 34, 35]. In Australia, S19A approvals allow the temporary supply of overseas‐registered medicines when local supply is unavailable; however, structural and behavioural barriers may constrain rapid uptake [5]. Factors such as prescriber familiarity, patient preference, distribution logistics, and residual supply limitations likely contributed to the limited utilisation observed, even once subsidised access was established [5]. Moreover, given that overseas products are generally more expensive to obtain, pharmacies may choose to keep limited S19A stock on hand in anticipation that PBS‐listing will be removed once a shortage is resolved. Anecdotally, most pharmacies prefer to order S19As on a case‐by‐case basis (informal communication).

### 4.4 Broader Implications

Increasing ADHD medicine use alongside supply instability highlights vulnerability in high‐demand therapeutic areas.

The divergence between continued growth in overall ADHD medicine dispensing and instability in methylphenidate utilisation highlights the vulnerability of high‐demand therapeutic areas to supply disruption. As ADHD diagnosis and treatment continue to expand, ensuring supply resilience for stimulant medicines becomes increasingly important. Policy responses may require system‐level strategies beyond temporary importation, including improved supply chain resilience, transparency and demand forecasting. Practical strategies that could be assessed for feasibility in the future include enhancing visibility across pharmacies regarding stock‐on‐hand, particularly for PBS medicines, to ensure that stock is spread equitably across Australia for people to access medicines. Additionally, an expedited PBS listing pathway for S19A product is also recommended, particularly if anticipatory approval of S19A product is not viable as we previously recommended in a separate analysis of the S19A pathway and its ability to mitigate shortages [5]. While there are strategies such as Serious Scarcity Substitution Instruments that allow for product substitutions during shortages without prior approval of a medical practitioner [36], enhanced evidence‐based guidance on therapeutic alternatives could be concurrently issued to medical practitioners and pharmacists at the same time to facilitate potential substitutions when required.

### 4.5 Strengths and Limitations

Integration of the TGA shortage reports enabled temporal comparison between utilisation changes and formally notified supply disruptions. Quantifying S19A overseas import products allowed for assessment of the population‐level contribution of this regulatory mechanism during the shortage period.

Several limitations should, however, be considered. We were unable to determine whether observed fluctuations in methylphenidate utilisation represented person‐level treatment discontinuation, switching between medicines, stockpiling, dose adjustment or changes in adherence. The unavailability of individual person‐level data precluded assessment of treatment continuity or patient outcomes. Moreover, the use of the DDD in this study is limited by the fact that methylphenidate is used in children, adolescents and adults and dosing varies by age, formulation and clinical indication. Therefore, the DDD may not accurately represent true use across the broad population of people who consume this medicine. The dataset captures subsidised dispensings only and does not include private prescriptions supplied outside of the PBS. Although stimulant medicines for ADHD are predominantly supplied via the PBS in Australia, private supply (cost per pack is approximately $30‐60AUD for methylphenidate; increasing price with increasing strength of tablets/capsules) may lead to some underestimation of total utilisation particular for patients who do not meet PBS criteria for specific ADHD medicines [10]. Moreover, the cost of non‐PBS S19A methylphenidate products was anecdotally reported to be in excess of $100 per month's supply (informal communication and viewing of pharmacy retail websites). To our knowledge, there is no literature‐based estimate of private‐prescription volume for methylphenidate; however, approximately 15% of the total prescription market in Australia is due to private (non‐PBS) supply of medicines [37]. Finally, the study period extends to March 2026 which was the latest available data at the time the study was conducted. Many of the reported shortages are still ongoing outside of the study period and may evolve beyond the observation window.

## 5 Conclusion

ADHD medicine dispensing increased substantially in Australia between 2020 and 2026. From late 2024 onward, methylphenidate utilisation showed marked fluctuation, coinciding with widespread and prolonged supply disruptions affecting multiple strengths and formulations. PBS‐listed S19A products contributed minimally to overall PBS dispensing during the main methylphenidate shortage period, likely due to delays in PBS listing limiting uptake. As demand for ADHD pharmacotherapy continues to rise, strengthened strategies are needed to improve supply resilience and responsiveness.

## Funding

The authors have nothing to report.

## Ethics Statement

This study used publicly and freely available aggregate dispensing data; therefore, ethics approval was not required.

## Conflicts of Interest

The authors declare no conflicts of interest.

## Supporting information


**Table S1:** PBS item code, drug name, anatomical therapeutic classification code (ATC) and Australian Register of Therapeutic Goods (ARTG) or Section 19A (S19A) classification.

**Data S1:** RECORD‐PE Checklist.

## Data Availability

Data for this study are freely and publicly available at: https://www.pbs.gov.au/info/statistics/dos‐and‐dop/dos‐and‐dop.
