# Supplementary material for: Temporal Changes in Dispensing of Methylphenidate During Attention Deficit Hyperactivity Disorder Shortages in Australia: An Interrupted Time Series and Descriptive Analysis of National Dispensing Patterns
Source: Pharmacoepidemiol Drug Saf. 2026 Sep 8;35(9):e70475. doi: 10.1002/pds.70475 (PMC13551630; doi:10.1002/pds.70475)
Supplement: Supplementary file 1 [file PDS-35-e70475-s001.docx]

**Supplementary file:**

Supplementarty table 1: PBS item code, drug name, anatomical therapeutic classification code (ATC) and Australian Register of Therapeutic Goods (ARTG) or Section 19A (S19A) classification.

| PBS item code | Drug | ATC Code | ARTG or S19A |
| --- | --- | --- | --- |
| 09092M | ATOMOXETINE Capsule 10 mg (as hydrochloride) | N06BA09 | ARTG |
| 09290Y | ATOMOXETINE Capsule 100 mg (as hydrochloride) | N06BA09 | ARTG |
| 09093N | ATOMOXETINE Capsule 18 mg (as hydrochloride) | N06BA09 | ARTG |
| 09094P | ATOMOXETINE Capsule 25 mg (as hydrochloride) | N06BA09 | ARTG |
| 09095Q | ATOMOXETINE Capsule 40 mg (as hydrochloride) | N06BA09 | ARTG |
| 09096R | ATOMOXETINE Capsule 60 mg (as hydrochloride) | N06BA09 | ARTG |
| 09289X | ATOMOXETINE Capsule 80 mg (as hydrochloride) | N06BA09 | ARTG |
| 01165H | DEXAMFETAMINE Tablet containing dexamfetamine sulfate 5 mg | N06BA02 | ARTG |
| 11440D | GUANFACINE 3 mg (as Tablet hydrochloride) | N06B | ARTG |
| 11452R | GUANFACINE Tablet 1 mg (as hydrochloride) | N06B | ARTG |
| 11451Q | GUANFACINE Tablet 2 mg (as hydrochloride) | N06B | ARTG |
| 11441E | GUANFACINE Tablet 4 mg (as hydrochloride) | N06B | ARTG |
| 11884L | LISDEXAMFETAMINE Capsule containing lisdexamfetamine dimesilate 20 mg | N06BA12 | ARTG |
| 11898F | LISDEXAMFETAMINE Capsule containing lisdexamfetamine dimesilate 40 mg | N06BA12 | ARTG |
| 10474G | LISDEXAMFETAMINE Capsule containing lisdexamfetamine dimesilate 50 mg | N06BA12 | ARTG |
| 11897E | LISDEXAMFETAMINE Capsule containing lisdexamfetamine dimesilate 60 mg | N06BA12 | ARTG |
| 10492F | LISDEXAMFETAMINE Capsule containing lisdexamfetamine dimesilate 70 mg | N06BA12 | ARTG |
| 10486X | LISDEXAMFETAMINE Capsule containing lisdexamfetamine dimesilate30 mg | N06BA12 | ARTG |
| 02276T | METHYLPHENIDATE Capsule containing methylphenidate hydrochloride 20 mg (modified release) | N06BA04 | ARTG |
| 02280B | METHYLPHENIDATE Capsule containing methylphenidate hydrochloride 30 mg (modified release) | N06BA04 | ARTG |
| 02283E | METHYLPHENIDATE Capsule containing methylphenidate hydrochloride 40 mg (modified release) | N06BA04 | ARTG |
| 03440C | METHYLPHENIDATE Capsule containing methylphenidate hydrochloride 10 mg (modified release) | N06BA04 | ARTG |
| 12116Q | METHYLPHENIDATE Capsule containing methylphenidate hydrochloride 60 mg (modified release) | N06BA04 | ARTG |
| 15003P | METHYLPHENIDATE Capsule containing methylphenidate hydrochloride 60 mg (modified release) (s19A) | N06BA04 | S19A |
| 15016H | METHYLPHENIDATE Capsule containing methylphenidate hydrochloride 20 mg (modified release) (s19A) | N06BA04 | S19A |
| 15026W | METHYLPHENIDATE Capsule containing methylphenidate hydrochloride 10 mg (modified release) (s19A) | N06BA04 | S19A |
| 15046X | METHYLPHENIDATE Capsule containing methylphenidate hydrochloride 30 mg (modified release) (s19A) | N06BA04 | S19A |
| 02387P | METHYLPHENIDATE Tablet containing methylphenidate hydrochloride 18 mg (extended release) | N06BA04 | ARTG |
| 08839F | METHYLPHENIDATE Tablet containing methylphenidate hydrochloride 10 mg | N06BA04 | ARTG |
| 14857Y | METHYLPHENIDATE Tablet containing methylphenidate hydrochloride 18 mg (extended release) Concerta (Switzerland) (S19A) | N06BA04 | S19A |
| 14861E | METHYLPHENIDATE Tablet containing methylphenidate hydrochloride 18 mg (extended release) (S19A) | N06BA04 | S19A |
| 02172H | METHYLPHENIDATE Tablet containing methylphenidate hydrochloride 27 mg (extended release) | N06BA04 | ARTG |
| 14874W | METHYLPHENIDATE Tablet containing methylphenidate hydrochloride 27 mg (extended release) (S19A) | N06BA04 | S19A |
| 02388Q | METHYLPHENIDATE Tablet containing methylphenidate hydrochloride 36 mg (extended release) | N06BA04 | ARTG |
| 14858B | METHYLPHENIDATE Tablet containing methylphenidate hydrochloride 36 mg (extended release) (S19A) | N06BA04 | S19A |
| 14862F | METHYLPHENIDATE Tablet containing methylphenidate hydrochloride 36 mg (extended release) Concerta (Switzerland) (S19A) | N06BA04 | S19A |
| 02432B | METHYLPHENIDATE Tablet containing methylphenidate hydrochloride 54 mg (extended release) | N06BA04 | ARTG |
| 14853R | METHYLPHENIDATE Tablet containing methylphenidate hydrochloride 54 mg (extended release) (S19A) | N06BA04 | S19A |
| 14871Q | METHYLPHENIDATE Tablet containing methylphenidate hydrochloride 54 mg (extended release) Concerta (Switzerland) (S19A) | N06BA04 | S19A |
